# Sequential occurrence of thrombotic thrombocytopenic purpura, essential thrombocythemia, and idiopathic thrombocytopenic purpura in a 42-year-old African-American woman: a case report and review of the literature

**DOI:** 10.1186/1752-1947-6-93

**Published:** 2012-04-03

**Authors:** Mirna H Farhat, Philip Kuriakose, Michael Jawad, Amr Hanbali

**Affiliations:** 1 Hematology/Oncology, Henry Ford Hospital, 2799 West Grand Boulevard, Detroit, MI 48202, USA; 2 Biology Department, University of Michigan, 4901 Evergreen Road, Dearborn, MI 48128, USA

## Abstract

**Introduction:**

Thrombotic thrombocytopenic purpura and idiopathic thrombocytopenic purpura are two well recognized syndromes that are characterized by low platelet counts. In contrast, essential thrombocythemia is a myeloproliferative disease characterized by abnormally high platelet numbers.

The coexistence of thrombotic thrombocytopenic purpura and idiopathic thrombocytopenic purpura in a single patient has been reported in the literature on a few occasions. However, having essential thrombocythemia complicating the picture has never been reported before.

**Case presentation:**

We present a case where thrombotic thrombocytopenic purpura, essential thrombocythemia, and idiopathic thrombocytopenic purpura were diagnosed in a 42-year-old African-American woman in the space of a few years; we are reporting this case with the aim of drawing attention to this undocumented occurrence, which remains under investigation.

**Conclusions:**

As the three conditions have different natural histories and require different treatment modalities, it is important to recognize that these diseases may be seen sequentially. This case emphasizes the importance of reviewing peripheral blood smears for evaluation of thrombocytopenia and bone marrow aspirations for diagnosis of thrombocythemia in order to reach an accurate diagnosis and tailor therapy accordingly. Moreover, this case demonstrates the variability and complexity of platelet disorders. This occurrence of three different types of platelet disorders in one patient remains a pure observation on our part; regardless, this does raise the possibility of a common underlying, as yet undiscovered, pathophysiology that could explain the phenomenon.

## Introduction

Thrombotic thrombocytopenic purpura (TTP) and idiopathic thrombocytopenic purpura (ITP) are two well recognized syndromes that are characterized by low platelet counts. In contrast, essential thrombocythemia (ET) is a myeloproliferative disease characterized by abnormally high platelet numbers.

The coexistence of TTP and ITP in a single patient has been reported in the literature on a few occasions. However, having ET complicating the picture has never been reported before. We present a case where TTP, ET, and ITP were diagnosed in the same patient in a span of a few years. The aim of this report was to draw attention to this undocumented occurrence, which remains under investigation.

## Case presentation

**Figure 1:**
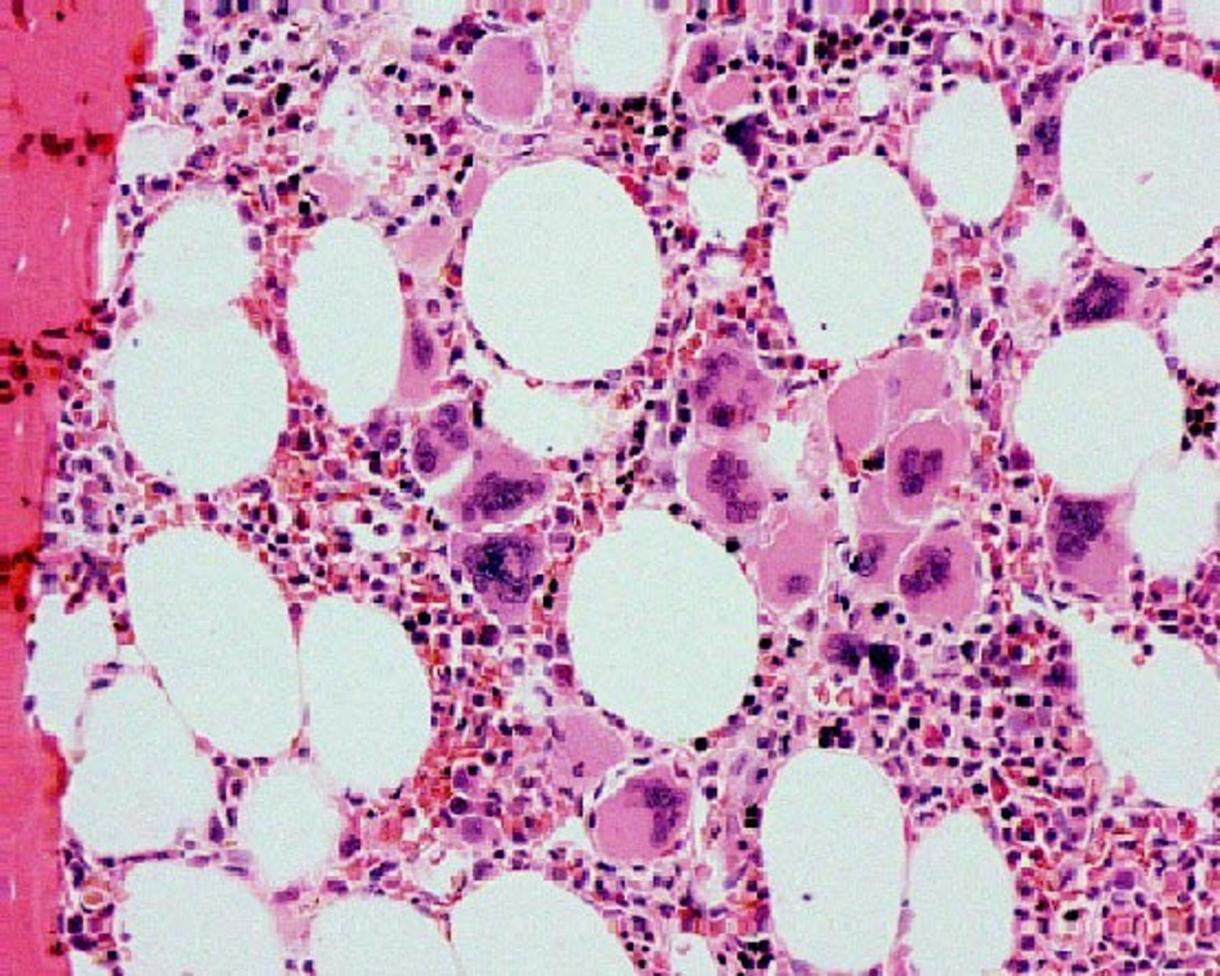
**Bone marrow biopsy in essential thrombocytosis showing increased megakaryocytes**.

A 42-year-old African-American woman was diagnosed in 1994 as having TTP after she presented to our facility with slurring of speech, left upper extremity weakness and a platelet count of 34,000 cells/mm^3^. She had a hemoglobin level of 9.7 g/dL, creatinine level of 0.9 mg/dL, and a peripheral smear showed schistocytes. Our patient was treated with plasmapheresis and vincristine and her hemolysis cleared and platelet count steadily increased. She was followed up closely, with her platelet count remaining in the normal range until March 2001, when she presented with a platelet count of 1,158,000 cells/mm^3 ^complicated by purple toe syndrome. A bone marrow biopsy showed fibrosis with 80% cellularity and clustering of megakaryocytes compatible with ET (Figure 1). Cytogenetic tests were negative for other myeloid disorders. Janus kinase 2 (JAK2) mutation testing was still not available at that time; however, our patient tested positive for this in subsequent years, which was in agreement with the bone marrow biopsy findings of TTP. Our patient was started on aspirin and anagrelide and her platelet count decreased to 700,000 cells/mm^3^. However, anagrelide was discontinued in May 2001 secondary to peripheral edema causing lower extremity discomfort and our patient was started on hydroxycarbamide 500 mg daily instead. Her platelet count ranged from 270,000 cells/mm^3 ^to 726,000 cells/mm^3 ^from March to October 2001. However, in October 2001 and while still on the same dose of hydroxycarbamide, her platelet count suddenly dropped down to 12,000 cells/mm^3^. Hydroxycarbamide was immediately stopped. Initially, TTP was suspected but her hemoglobin, bilirubin and lactate dehydrogenase (LDH) levels were normal. The results of a peripheral smear were negative for schistocytes. A spleen examination was also normal. A bone marrow biopsy was performed to rule out TTP versus bone marrow suppression, but it showed abundant megakaryocytes and absence of stainable iron. Our patient was transfused with 12 units of platelets, and her platelet count was 90,000 cells/mm^3 ^post-transfusion, but in less than 12 hours the platelet level went down to 25,000 cells/mm^3^. These findings raised the suspicion of ITP. She was started on dexamethasone 40 mg for four days with no improvement in platelet count and on day four, intravenous immunoglobulin was introduced and her platelets recovered to 290,000 cells/mm3, which confirmed the diagnosis of ITP.

The results of a subsequent out-patient evaluation were negative for anti-nuclear antibodies (ANA), C3, C4, and Human immunodeficiency virus (HIV). Our patient was not pregnant at any time during her illness.

## Discussion

TTP is a multisystem disorder characterized by deposition of intra-vascular platelet microthrombi, causing consumption thrombocytopenia, microangiopathic hemolytic anemia, renal abnormalities, neurologic disturbances and fever. ITP is characterized by low platelet count with otherwise normal results on complete blood count and peripheral blood smear. It is an isolated thrombocytopenia with no other underlying etiology. Contrary to the two above-mentioned disorders, ET is a myeloproliferative disease characterized by sustained and unexplained proliferation of megakaryocytes leading to increased platelet count, often in excess of 1,000,000/mm^3^.

ITP is a disease caused by autoantibodies to platelets. The antigenic target in most patients appears to be the platelet glycoprotein IIb/IIIa complex [1] Platelets with antibodies on their surface are trapped in the spleen, where they are efficiently removed by splenic macrophages. The origin of these antibodies is not known. They may be directed towards the viral antigens and then cross-react with platelet antigens. Recent observations have documented that a deficiency of a Von Willebrand factor (VWF)-cleaving protease, termed ADAMTS13 ('a disintegrin and metalloproteinase with a thrombospondin type 1 motif, member 13'), that normally cleaves hyper-reactive unusually large VWF multimers into smaller and less adhesive VWF forms, may be responsible for many cases of TTP [2]. A mutation in JAK2 kinase (V617F) was found to be associated with essential thrombocytosis [3]. A diagnosis of ET is made when a patient has an elevated platelet count, an increased number of megakaryocytes in the bone marrow with no identifiable underlying abnormality known to cause thrombocytosis and the absence of findings suggestive of a different myeloproliferative disorder.

TTP transformation to ITP has been described previously in the literature [4,5] as has their coexistence in a single patient with HIV [6], in post-partum states [7], and in patients with systemic lupus erythematosus (SLE) [8], all of which were absent in our patient.

A review performed by Baron *et al. *in 2001 identified 11 cases in the literature that developed both ITP and TTP concomitantly or sequentially [5]. In the 11 described cases, two were male, nine were female, and their ages ranged from 14 to 62 years. Associated medical conditions were autoimmune disorders such as hypothyroidism, SLE, rheumatoid arthritis and Sjogren's disease. The two conditions, TTP and ITP, were noted to occur days to years apart, the longest duration being two and a half years.

None of the patients previously reported to have TTP and ITP had essential thrombocythemia as part of their clinical course.

Our patient had no evidence of HIV infection, had no associated autoimmune disease and was not pregnant. Moreover, the increased platelet count in our patient was of primary origin, was not post-splenectomy and was not reactive to drugs as suggested by the findings on bone marrow biopsy. ITP occurred seven years after the diagnosis of TTP.

The fact that both immune and thrombotic thrombocytopenic purpura occur with increased frequency among persons with systemic lupus erythematosus, HIV or pregnancy supports the hypothesis that some pathophysiologic factors are shared. These include: circulating antibodies or antigen-antibody complexes caused by the primary autoimmune disorder and inducing endothelial dysfunction [9]; platelet damage by TTP and production of autoantibodies [4]; deficiency of Von-Willebrand factor cleaving protease activity [10] or autoantibody against VWF cleaving protease [11]; molecular mimicry or redundancy of the immune system, also known as the kaleidoscope of immunity, which is the co-occurrence of various autoimmune diseases within an individual [12].

The association of TTP and ITP in the same patient supports the notion that TTP and ITP share similar pathogenetic mechanism; however, there is no known common factor implicated in the etiology of all three platelet disorders.

## Conclusions

As the three conditions have different natural histories and require different treatment modalities, it is important to recognize that these diseases may be seen sequentially. It is crucial to emphasize that once a diagnosis of ITP is made, all future thrombocytopenic presentations should still have a blood film review and clinical assessment of the patient. It also highlights the importance of reviewing peripheral blood smears for evaluation of thrombocytopenia and bone marrow aspirations for diagnosis of thrombocythemia in order to reach an accurate diagnosis and tailor therapy accordingly. Finally, this case demonstrates the variability and complexity of platelet disorders. This occurrence of three different types of platelet disorder in one patient remains a pure observation on our part; regardless, this does raise the possibility of a common underlying, as yet undiscovered, pathophysiology that could explain the phenomenon.

## Consent

Written informed consent was obtained from the patient for publication of this case report and accompanying images. A copy of the written consent is available for review by the Editor-in-Chief of this journal.

## Competing interests

The authors declare that they have no competing interests.

## Authors' contributions

MF and MJ were major contributors in writing the manuscript. AH and PK reviewed and edited the manuscript. All authors read and approved the final manuscript.
